# A *CACNA1C* Variant Associated with Reduced Voltage-Dependent Inactivation, Increased Ca_V_1.2 Channel Window Current, and Arrhythmogenesis

**DOI:** 10.1371/journal.pone.0106982

**Published:** 2014-09-03

**Authors:** Jessica A. Hennessey, Nicole J. Boczek, Yong-Hui Jiang, Joelle D. Miller, William Patrick, Ryan Pfeiffer, Brittan S. Sutphin, David J. Tester, Hector Barajas-Martinez, Michael J. Ackerman, Charles Antzelevitch, Ronald Kanter, Geoffrey S. Pitt

**Affiliations:** 1 Department of Medicine/Cardiology, Duke University School of Medicine, Durham, North Carolina, United States of America; 2 Department of Pharmacology and Cancer Biology, Duke University School of Medicine, Durham, North Carolina, United States of America; 3 Department of Neurobiology, Duke University School of Medicine, Durham, North Carolina, United States of America; 4 Department of Pediatrics, Duke University School of Medicine, Durham, North Carolina, United States of America; 5 Department of Pediatrics/Cardiology, Duke University School of Medicine, Durham, North Carolina, United States of America; 6 Departments of Molecular Pharmacology and Experimental Therapeutics, Windland Smith Rice Sudden Death Genomics Laboratory, Mayo Clinic, Rochester, Minnesota, United States of America; 7 Departments of Internal Medicine and Pediatrics & Adolescent Medicine/Divisions of Cardiovascular Diseases and Pediatric Cardiology, Mayo Clinic, Rochester, Minnesota, United States of America; 8 Department of Pediatrics, Virginia Tech Carilion School of Medicine, Roanoke, Virginia, United States of America; 9 Masonic Medical Research Laboratory, Utica, New York, United States of America; National University of Singapore, Singapore

## Abstract

Mutations in *CACNA1C* that increase current through the Ca_V_1.2 L-type Ca^2+^ channel underlie rare forms of long QT syndrome (LQTS), and Timothy syndrome (TS). We identified a variant in *CACNA1C* in a male child of Filipino descent with arrhythmias and extracardiac features by candidate gene sequencing and performed functional expression studies to electrophysiologically characterize the effects of the variant on Ca_V_1.2 channels. As a baby, the subject developed seizures and displayed developmental delays at 30 months of age. At age 5 years, he displayed a QTc of 520 ms and experienced recurrent VT. Physical exam at 17 years of age was notable for microcephaly, short stature, lower extremity weakness and atrophy with hyperreflexia, spastic diplegia, multiple dental caries and episodes of rhabdomyolysis. Candidate gene sequencing identified a G>C transversion at position 5731 of *CACNA1C* (rs374528680) predicting a glycine>arginine substitution at residue 1911 (p.G1911R) of Ca_V_1.2. The allele frequency of this variant is 0.01 in Malays, but absent in 984 Caucasian alleles and in the 1000 genomes project. In electrophysiological analyses, the variant decreased voltage-dependent inactivation, thus causing a gain of function of Ca_V_1.2. We also observed a negative shift of V_1/2_ of activation and positive shift of V_1/2_ of channel inactivation, resulting in an increase of the window current. Together, these suggest a gain-of-function effect on Ca_V_1.2 and suggest increased susceptibility for arrhythmias in certain clinical settings. The p.G1911R variant was also identified in a case of sudden unexplained infant death (SUID), for which an increasing number of clinical observations have demonstrated can be associated with arrhythmogenic mutations in cardiac ion channels. In summary, the combined effects of the *CACNA1C* variant to diminish voltage-dependent inactivation of Ca_V_1.2 and increase window current expand our appreciation of mechanisms by which a gain of function of Ca_V_1.2 can contribute to QT prolongation.

## Introduction

Few arrhythmogenic mutations in *CACNA1C*, which encodes the pore-forming α_1C_ subunit of the Ca_V_1.2 L-type Ca^2+^ channel have been reported in long QT syndrome (LQTS), an inherited or acquired arrhythmia characterized by an abnormally long electrocardiographic QT interval, which reflects an underlying prolonged ventricular cardiomyocyte action potential caused by the specific biophysical consequences of the underlying mutation. Much more common are mutations in K^+^ channels and Na^+^ channels [1], [2], reported almost a decade before Ca^2+^ channel arrhythmia mutations were first described in Timothy Syndrome (TS, OMIM #601005, long QT syndrome 8). In addition to prolonged intervals, TS is also associated with many extracardiac phenotypes including syndactyly, cognitive delay, and craniofacial abnormalities [3].

The first TS-associated mutation identified in *CACNA1C* was p.G406R in the alternatively spliced exon 8a of *CACNA1C.* Functional characterization of p.G406R revealed a marked reduction in voltage-dependent inactivation (VDI); the consequent increase in Ca^2+^ influx prolongs the cardiac action potential, and thus the QT interval, and can generate early afterdepolarizations capable of triggering life-threatening arrhythmias [3]–[5]. A variant of TS (denoted TS2) was identified in two patients who had either a G406R mutation or a G402S mutation in the predominant exon 8 and who presented with a subset of the phenotypes found in the original TS cohort [4]. Like the G406R mutation in the exon 8a, the G406R/G402S mutations in exon 8 also diminish VDI [4].

Additionally, several *CACNA1C* mutations have recently been identified in patients presenting with LQTS but without any obvious extracardiac manifestations [6], [7]. Two of these, P857R and R858H, exert a gain-of-function effect through an apparently VDI-independent mechanism. Instead, the P857R mutation leads to an increase of Ca_V_1.2 on the surface membrane, thereby increasing the resulting Ca^2+^ current [6]. The R858H mutation also increases current density, but the underlying mechanism has not yet been investigated [7]. In contrast, a A582D mutation slowed channel inactivation [7]. A total of six additional mutations were identified in a 102- and 278-patient cohort of previously genotype-negative LQTS patients in whom extracardiac phenotypes were absent [6], [7], suggesting that arrhythmogenic variants in *CACNA1C* are perhaps more common than previously expected.

Several polymorphisms have reported in *CACNA1C*, but the functional significance of most of these is not yet known. Relatively common polymorphisms (variably defined by a minor allele frequency ≥0.01) are usually expected to be benign because of their survival in the face of evolutionary pressure. Recently, however, polymorphisms in several LQTS loci have been shown to increase arrhythmia susceptibility in certain clinical settings. Perhaps best characterized is p.S1103Y in *SCN5A* (rs7626962), a LQTS locus that encodes the cardiac Na_V_1.5 Na^+^ channel. This variant is found almost exclusively in people of African descent and is present in 13% of African Americans. It was first discovered in a patient with drug-induced LQTS. Electrophysiological characterization revealed only minimal effect on Na^+^ currents through Na_V_1.5, a small increase in the “late” Na^+^ current. In the setting of drug-induced HERG K^+^ channel blockade, however, the small increase in the late Na^+^ current is sufficient to prolong the cardiac action potential, thereby providing a substrate for life-threatening arrhythmias [8]. Thus, p.S1103Y does not cause arrhythmias on its own, but increases arrhythmia propensity from a “second hit”. Indeed multiple studies have found p.S1103Y to be a marker for increased arrhythmia risk from provocative stimuli such as cardiomegaly [9] and heart failure [10]. Here, we characterize a *CACNA1C* variant (rs374528680), p.G1911R, in a subject of Filipino ancestry who presented with LQTS, ventricular arrhythmias, and a multitude of extracardiac phenotypes. This minor allele frequency of this variant is 0.01 in Malay subjects, but has not been reported in other ethnic groups. Because sudden unexpected infant death (SUID) has been linked to mutations in LQTS loci–and particularly associated with S1103Y [11], [12], suggesting that physiologic or metabolic changes in the context of a pro-arrhythmic variant may increase the risk for life-threatening events–we also looked for the presence of this variant among a SUIDS cohort, in which the p.G1911R variant was subsequently identified in an additional subject. Our biophysical analysis shows that p.G1911R variant reduces Ca_V_1.2 VDI and affects the window current. These analyses expand our understanding of how variants in *CACNA1C* can lead to an arrhythmogenic phenotype and suggest that rs374528680 encoding p.G1911R in Ca_V_1.2 may increase arrhythmia susceptibility under certain conditions.

## Methods

### Sequencing

Blood was collected from the subject and his mother after obtaining written informed consent from the patient’s mother. The sequencing was approved by the Institutional Review Board at the Masonic Medical Research Laboratory. Genomic DNA was extracted from peripheral blood lymphocytes using a commercial kit (Puregene, Gentra Systems, Inc. Minneapolis, MN). Libraries were constructed using Custom Ion Ampliseq (Life Technologies Carlsbad, CA) to amplify all exons and intron borders of a targeted next generation panel comprising all genes known to be associated with ion channelopathies on a Veriti Thermal Cycler (Life Technologies Carlsbad, CA). Amplified samples were uniquely barcoded and pooled equal-molar for sequencing on an Ion Personal Genome Machine (PGM) (Life Technologies Carlsbad, CA). Ion Torrent Suite software was used to map the sequencing reads to the DNA reference sequence [hg19] and identify variants through the VariantCaller plugin as well as the IonReporter analysis tool. Run quality was assessed using the Coverage Analysis plugin to report average depth of coverage, uniformity of coverage, and percent on target as well as other metrics. Missense variations with an allele frequency of <0.5% in the 1000 genomes database were classified as variants. Common variants identified were H558R in *SCN5A*, G38S in *KCNE1* and A1073V in *SCN10A*.

The *CACNA1C* variant uncovered was confirmed using Sanger sequencing. PCR products were purified with a commercial enzyme (ExoSAP- IT, USB, Cleveland, OH) and directly sequenced from both directions using Big Dye Terminator 3.1 chemistry on an Applied Biosystems 3730 DNA Analyzer (Life Technologies Carlsbad, CA). All other genes were negative, including the other Ca^2+^ channel auxiliary subunit genes *CACNB1-4* and *CACNA2D1*.

### Variant-specific analysis

The p.G1911R variant-specific analysis was completed using denaturing high performance liquid chromatography (DHPLC; WAVE DNA Fragment Analysis System, Transgenomic Inc, Omaha, NE) and direct DNA sequencing (ABI Prism 377; Applied Biosystems Inc., Foster City, CA) on 292 unrelated SUID cases (114 female infants, 178 male infants; average age, 2.9±1.9 months; range, 6 hours - 12 months) that were submitted to the Mayo Clinic Windland Smith Rice Sudden Death Genomics Laboratory for post-mortem genetic testing. To be defined as SUID, the death of the infant under age one year had to be sudden, unexpected, and unexplained following a comprehensive medico-legal autopsy [13]. Infants whose deaths were due to asphyxia or specific disease were excluded. This study was approved by Mayo Clinic’s Institutional Review Board as an anonymous study. As such, only limited medical information was generally available, including sex, ethnicity, and age at death. Time of day, medication use, and position at death were not available. Patient records/information were fully anonymized and de-identified prior to analysis.

### Site-directed mutagenesis and electrophysiology

Human *CACNA1C* (Accession no. NM_000719) was mutagenized using site-directed mutagenesis (Agilent), following the manufacturer’s instructions using the following primers: 5′ CAGAAGGACCGA**C**GCGGAGACATCTCTC 3′ (forward); 5′ GAGAGATGTCTCCGC**G**TCGGTCCTTCTG 3′ (reverse). The underlined and bolded nucleotide indicates the variant. HEK293T cells were transfected with plasmids expressing human *CACNA1C* (3 µg, wild-type or G1911R), *CACNB2B* (1.5 µg), and *CACNA2D* (2.2 µg) and green fluorescent protein (GFP, 0.2 µg) in 60 mm cell culture plates at a confluency of 40% to 60% using Lipofectamine 2000 (Life Technologies). The day before planned recordings, the cells were resuspended in a single-cell suspension and plated onto glass coverslips. L-type Ca^2+^ channel current was recorded at room temperature (∼25°C) in cells expressing GFP using whole-cell patch clamp with an Axopatch 200B amplifier and pCLAMP 10 acquisition software. Electrodes with a resistance of 2 to 3 MΩ. Cells were bathed in normal Tyrode solution containing (in mM, from Sigma): NaCl 140, KCl 5.4, CaCl_2_ 1, MgCl2 1, HEPES 5, glucose 10, pH 7.3 adjusted with NaOH. Once the cell was ruptured, solution was quickly changed to recording solution containing (in mM, from Sigma): 124 NaCl, 20 TEA-Cl, 1 MgCl_2_, 10 BaCl_2_, 5 HEPES, and 10 glucose, pH 7.3, with NaOH (300–310 mOsm). The internal solution contained (in mM) 115 CsCl, 20 TEA-Cl, 1 CaCl_2_, 2 MgCl_2,_ 10 HEPES, and 2 Mg-ATP, pH 7.3, with CsOH (290–300 mOsm). Series resistance was at least 80% compensated and capacitance was compensated. To measure current density and voltage-dependence of activation, cells were held at –80 mV and stepped from –80 mV to +40 mV for 400 ms in 10 mV increments. Current density (pA/pF) was calculated by dividing the peak current for a given voltage-step by capacitance. Conductance (g) was calculated using the equation *g = I/(Vm–Vr),* then normalized to the maximum to give relative conductance (g/g_max_). To determine V_1/2_ and *k*, the curve was fit to the Boltzmann function, G/*G*_max_ = *A*_1_ *+(*1*–A*_1_)/(1+exp[(*V*_1/2_–*V*_m_)/*k*]). Steady-state inactivation was performed with a standard two-pulse protocol. From a holding potential of –80 mV, V_m_ was stepped for 3 s to a preconditioning potential (–80 mV to +20 mV; prepulse), followed by a 300 ms test pulse to 0 mV. Current density was normalized to the maximum and the curve was fit with the Boltzmann function, I/*I*_max_ = *A*_1_ *+(*1*–A*_1_)/(1+exp[(*V*_m_–*V*_1/2_)/*k*]).

### Statistical analysis

Results are presented as means ± standard error of the mean; statistical significance of differences between or among groups was assessed using Student’s t-test or one-way analysis of variance (ANOVA), respectively, and was set at P<0.05.

## Results

### Clinical Report

The proband was born at 42 weeks gestation to a healthy G4P4 adopted mother who believes she is of Mexican and American heritage and a father of Caucasian and Filipino descent. The couple was healthy and non-consanguineous. The adopted mother’s family history is unknown and the father’s family history was negative for cardiac arrhythmias or syndromic disorders. The mother smoked cigarettes during the pregnancy, which was uneventful except for significant bleeding during the second trimester that resolved during the third trimester, and required no intervention. Delivery was vaginal and uncomplicated. Birth weight was 3.6 kg (greater than 95th percentile) and length was 50.8 cm (50 to 75th percentile). No physical exam abnormalities were noted at this time.

The proband developed normally until age 30 months, when he began having seizures manifested as sudden staring, often with drooling or posturing, followed by a loss of consciousness. This was accompanied by growth and developmental delay with significant language and gait regression. At age 5 years, he experienced a seizure during which he was found to be in monomorphic ventricular tachycardia at a rate of 300 beats per minute. Electrocardiogram after resuscitation showed a prolonged QTc of 520 ms. Ventricular tachycardia was not inducible during subsequent electrophysiology testing. He received a transvenous implantable cardioverter defibrillator (ICD) and was put on propranolol without recurrence of arrhythmias or seizures for eight years. At the time of the initial ventricular tachycardia presentation, electrolyte levels were normal but creatine phosphokinase (CK) levels were elevated to 7000 IU and myoglobinuria was demonstrated. Electromyography was abnormal and consistent with a myopathic process.

**Figure 1:**
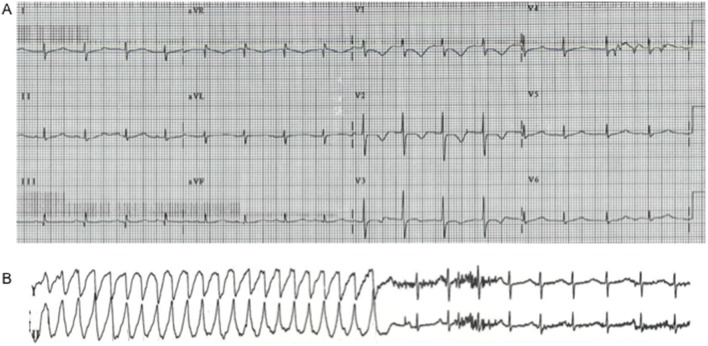
Long QT and ventricular tachycardia. A: Baseline electrocardiogram showing prolonged QT interval. The heart rate is 97. The QTc is 504 ms. The ECG was recorded at 25 mm/s. B: Leads I and II showing monomorphic ventricular tachycardia on an ECG rhythm strip recorded in the Emergency Department.

At age 12½ years the patient presented with a reported absence seizure and subsequent ICD discharge in the setting of hypokalemia. A baseline ECG showed a QTc of 504 ms (Figure 1A). Recurrent episodes of ventricular tachycardia occurred even after serum potassium was repleted (Figure 1B). The addition of atrial pacing significantly reduced the ventricular ectopy burden. CK levels were again elevated during this episode to above 5000 IU and skeletal muscle biopsy showed degenerating fibers and myophagocytosis. The histology and ultrastructure of muscle sample was otherwise unremarkable other than decreased mitochondrial complex I and III activity by enzymology assay and quantitative immunoblot. Physical exam at age of 17 years was notable for microcephaly, short stature, lower extremity weakness and atrophy with hyperreflexia, and multiple dental caries. He has malar prominence, flattened nasal bridge, small upper and lower jaws, protruding upper teeth, and micrognathia. No other significant congenital anomaly was noted.

Initial karyotype analysis reported a karyotype of 46, XY, t(6;12) (p21.3;q21.2). Subsequent chromosomal microarray analysis by array-based comparative genomic hybridization (using the Affymetrix 6.0 SNP array platform) demonstrated genomic loss involving chromosome 6p21.1, including a 123 kilobase deletion in the *SUPT3H* gene. Testing of the asymptomatic mother revealed that the deletion is inherited from mother, but her karyotype was not obtained. Genetic testing for muscular dystrophies (*EMD*, *LMNA*, *CAV3*) and for Andersen-Tawil syndrome (*KCNJ2*) was negative, as were mitochondrial DNA mutation studies.

### Identification of a *CACNA1C variant*

**Figure 2:**
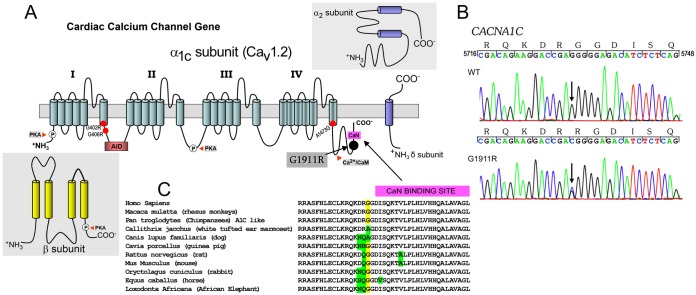
Location of the p.G1911R variant in the α_1C_ subunit of Ca_V_1.2. A: Schematic of the Ca_V_1.2 channel pore-forming α_1C_ subunit and the auxiliary α_2_δ and β subunit. The p.G1911R variant is in the C-terminus close to the calcineurin (CaN) binding site. The location of other mutations in α_1C_ previously associated with TS are also indicated, including the most commonly reported p.G402R and p.G406R in the loop between DI and DII, and A1473G in the transmembrane segment 6 in the DIV. AID, α1 subunit interacting domain. B: Sanger sequencing of a normal control (WT) and the patient’s DNA showing the p.G1911R variant (arrow). C: Amino acid alignment shows conservation among species of glycine (G) at position 1911 also showing the CaN binding domain.

We examined all exons and intron borders of a targeted next generation panel comprising all genes known to be associated with ion channelopathies. Common variants identified were H558R in *SCN5A*, G38S in *KCNE1* and A1073V in *SCN10A*. No rare nonsynonymous variants were found except c.5731G>C (accession # NM_000719), predicting a glycine to arginine substitution at amino acid 1911 (p.G1911R) (Figure 2A–B). This was absent in his mother and more than 200 Caucasian healthy controls (400 reference alleles). The variant is also absent in the 1000 genomes project [14] and in the NHLBI Exome Sequencing Project (ESP) database (http://evs.gs.washington.edu/EVS/). DNA from the subject’s father was not available for testing. Following variant specific analysis of 292 cases of SUID, p.G1911R was identified in a one female who died suddenly and unexpectedly at one month of age. Subsequent to our initial analyses, the p.G1911R variant was reported in dbSNP (rs374528680). The studied population was Malays, in whom the minor allele frequency was 0.01 [personal communication, T.Y. Ying and L.P. Wong, National University of Singapore]. Our identification of the variant in the Filipino proband and in only 1 other allele within the 984 tested, in combination with the variant’s absence in the 1000 genomes project suggests that p.G1911R variant is rare outside of the Malay population. SIFT and PolyPhen-2 predictions are 0.35 and 0.286, respectively, but analysis of G1911 across multiple species shows that Gly at position 1911 is conserved among all species examined with the exception of marmoset and canine (Figure 2C).

### Functional analysis of mutant channel

**Figure 3:**
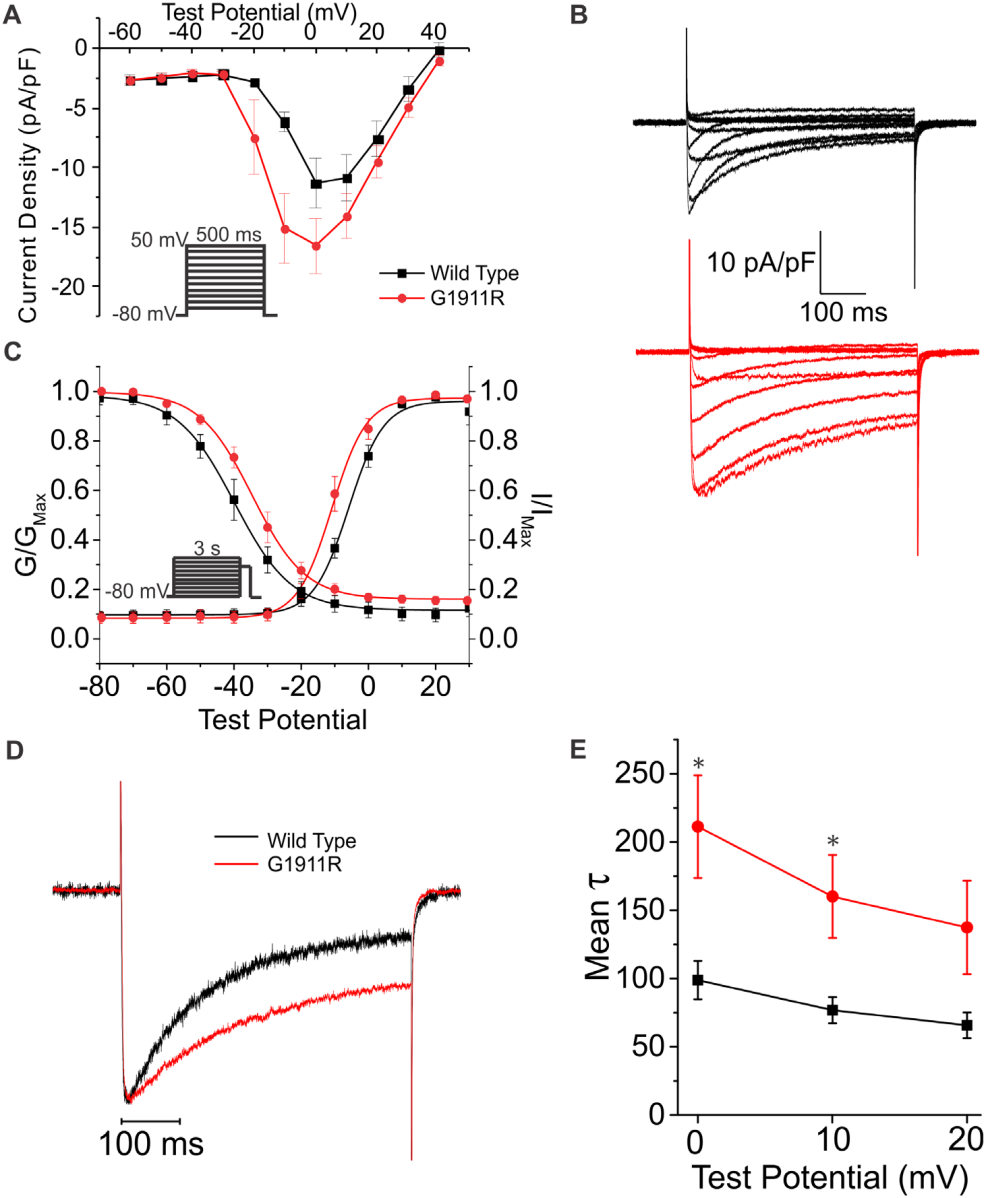
p.G1911R affects Ca_V_1.2 availability and VDI. A–B: Current-voltage relationship and representative current traces showing an increase in current density at more negative potentials. C: voltage-dependence of activation and steady-state inactivation curves showing a hyperpolarizing and depolarizing shift, respectively leading to increased availability and window current. D: p.G1911R decreases voltage-dependent inactivation as measured by fitting a single exponential and comparing the tau value. Summarized in E. See Table 1 for values. *p<0.05.

We performed functional analyses to determine whether p.G1911R affected channel function. We expressed wild-type (WT) or p.G1911R Ca_V_1.2 in HEK293T cells along with its accessory subunits β_2_ and α_2_δ. Using Ba^2+^ as the charge carrier to focus specifically upon VDI, we performed whole-cell patch clamp (Figure 3, Table 1). Current density appeared increased by the p.G1911R variant compared to WT (Figure 3A–B, Table 1), but was not statistically significant. Additionally, voltage-dependence of activation was shifted ∼–5 mV and steady-state inactivation shifted ∼+6 mV, thereby significantly increasing the window in which the channels are available (“window current”) compared to WT (Figure 3C, Table 1). Additionally, the tau of inactivation measured using a single exponential fit was larger for p.G1911R compared to WT over a range of voltages (Figure 3D–E, Table 1), showing a defect in VDI.

**Table 1:** Electrophysiological parameters for the wild type and p.G1911R mutant channel.

	WT	p.G1911R	P Value
Current Density (pA/pF)	–11.3±2.1 (12)	–16.5±2.3 (11)	0.118
V_1/2_ activation (mV)	–4.9±1.1 (12)	–9.7±1.7 (11)*	0.026
k activation	4.9±0.4 (12)	4.5±0.6 (11)	0.635
V_1/2_ inactivation (mV)	–39.2±1.7 (7)	–33.4±1.9 (9)*	0.048
k inactivation	7.6±0.9 (7)	6.7±0.3 (9)	0.282
A_1_ activation	0.10±0.02 (12)	0.09±0.02 (11)	0.784
A_1_ inactivation	0.11±0.03 (7)	0.16±0.01 (9)	0.101
tau inactivation at 0 mV (s)	98.9±14.0 (6)	211.3±37.6(9)*	0.036
tau inactivation at +10 mV (s)	76.8±9.5 (6)	160.1±30.4 (8)*	0.040
tau inactivation at +20 mV (s)	65.7±9.4 (6)	137.4±34.2 (6)	0.103

Numbers tested are in parentheses.

*p<0.05 vs WT.

## Discussion

We describe a child with a prolonged QT_C_ interval, life-threatening arrhythmias, seizures, developmental delay, microcephaly, short stature, lower extremity weakness and atrophy with hyperreflexia, spastic diplegia, multiple dental caries and episodes of rhabdomyolysis. Screening of arrhythmia associated genes revealed a G1911R variant in the *CACNA1C-*encoded L-type calcium channel α_1C_ subunit. Mutations within *CACNA1C* have been identified in LQTS, as well as Timothy syndrome, a disease characterized by QT prolongation and extracardiac abnormalities. Similarly, our case presented with QT prolongation, and some of the extracardiac phenotypic features overlap with TS. Some of the extracardiac features, however, are unique to this case, such as the microcephaly, short stature, and spastic diplegia.

The biophysical defects uncovered in functional testing of the p.G1911R mutant Ca_V_1.2 channel show a gain-of-function (increased window current and decreased VDI), both of which would increase Ca^2+^ influx during the cardiac action potential and lead to a longer action potential duration. This overall effect is consistent with the gain-of-function observed for previously characterized arrhythmogenic *CACNA1C* mutations in LQTS and TS patients [3], [4], [7], [15]. Therefore, we believe that the proband’s p.G1911R *CACNA1C* variant may contribute to the long QTc interval and arrhythmias based on the electrophysiological analysis of the variant in a heterologous expression system.

The mechanism by which the p.G1911R variant alters Ca_V_1.2 channel function is not known, but p.G1911R variant occurs in a highly conserved residue in close proximity to a calcineurin (CaN)-binding site, known to modulate voltage-dependent inactivation of the Ca_V_1.2 channel [16], [17]; thus, perturbation of the CaN binding site may alter its interaction with the channel, and thereby affect voltage-dependent interaction. The distal Ca_V_1.2 C-terminus, where the p.G1911R variant in is located, also plays an important role in channel inactivation. It has been proposed that the distal C-terminus contains an inhibitory peptide that, when cleaved or mutated, dramatically increases the conductance of the channel [18], suggesting that it may participate in VDI. In the context of the recently reported p.R1906Q variant in a patient with LQTS [6], this C-terminal region may become known as a new hotspot for arrhythmia-causing variants in *CACNA1C*.

Although we were unable to determine if the proband’s p.G1911R *CACNA1C* variant was paternally inherited from his phenotypically normal father, we believe that is possible, given that his father is Filipino and this p.G1911R appears to be enriched in Malays. Because this variant is relatively common in a specific ethnic population (minor allele frequency ∼0.01), we speculate that the variant may increase susceptibility to arrhythmogenesis, but the biophysical changes associated with the variant may not be sufficient to cause arrhythmias alone. Thus, p.G1911R in *CACNA1C* may be akin to p.S1103Y in *SCN5A*: providing an increased propensity towards arrhythmogenesis for a particular ethnic population under conditions of specific stimuli. It is thus intriguing that arrhythmia presentations in the proband occurred in clinical settings when other disturbances were present, such as a seizure associated with myoglobinuria or in the setting of hypokalemia. It is also interesting this *CACNA1C* variant is enriched in Malays, as the arrhythmogenic sudden unexpected nocturnal death syndrome is especially common in South East Asians [19]. In this context, the identification of a SUID case in which this variant was present may provide additional support for our hypothesis that p.G1911R in *CACNA1*C increases arrhythmia susceptibility. Whether p.G1911R in *CACNA1C* contributed to any of the other phenotypes displayed by the proband and previously associated with *CACNA1C* mutations in TS (e.g., facial dysmorphia and dental caries) is not known at this time.

## Conclusion

In summary, a p.G1911R variant in *CACNA1C* found in Malays results in a gain of function of Ca_V_1.2 current via a defect in VDI and increased Ca_V_1.2 window current, and was associated with an arrhythmogenic phenotype in the proband and with a case of SUID. This study offers new insight into the critical role of *CACNA1C* in cardiac rhythm.

## Supporting Information

Checklist S1 ARRIVE checklist.
(PDF)
